# Sustainable deployment of clinical prediction tools—a 360° approach to model maintenance

**DOI:** 10.1093/jamia/ocae036

**Published:** 2024-02-29

**Authors:** Sharon E Davis, Peter J Embí, Michael E Matheny

**Affiliations:** Department of Biomedical Informatics, Vanderbilt University Medical Center, Nashville, TN 37203, United States; Department of Biomedical Informatics, Vanderbilt University Medical Center, Nashville, TN 37203, United States; Department of Medicine, Vanderbilt University Medical Center, Nashville, TN 37232, United States; Department of Biomedical Informatics, Vanderbilt University Medical Center, Nashville, TN 37203, United States; Department of Medicine, Vanderbilt University Medical Center, Nashville, TN 37232, United States; Department of Biostatistics, Vanderbilt University Medical Center, Nashville, TN 37203, United States; Geriatric Research, Education, and Clinical Care, Tennessee Valley Healthcare System VA Medical Center, Veterans Health Administration, Nashville, TN 37212, United States

**Keywords:** algorithmovigilance, dataset shift, predictive analytics, model updating, performance drift, artificial intelligence

## Abstract

**Background:**

As the enthusiasm for integrating artificial intelligence (AI) into clinical care grows, so has our understanding of the challenges associated with deploying impactful and sustainable clinical AI models. Complex dataset shifts resulting from evolving clinical environments strain the longevity of AI models as predictive accuracy and associated utility deteriorate over time.

**Objective:**

Responsible practice thus necessitates the lifecycle of AI models be extended to include ongoing monitoring and maintenance strategies within health system algorithmovigilance programs. We describe a framework encompassing a 360° continuum of preventive, preemptive, responsive, and reactive approaches to address model monitoring and maintenance from critically different angles.

**Discussion:**

We describe the complementary advantages and limitations of these four approaches and highlight the importance of such a coordinated strategy to help ensure the promise of clinical AI is not short-lived.

As artificial intelligence (AI) continues to mature towards broad implementation within clinical systems, successful integration requires comprehensive, system-based approaches.^1^ We have seen hundreds of predictive models developed targeting important health outcomes, yet for any number of reasons, few are deployed in clinical tools. The advent of large language models has further generated a flurry of proposed applications whose implementation realities are yet to be determined. What we know, however, is that training accurate models is not enough to ensure those models can support decision-making or improve patient outcomes. Successful clinical AI tools must ensure algorithmic fairness and develop user trust. They must provide actionable information when and how that information best supports decision-making. They must consistently deliver clinical utility. Certainly, there is excitement in integrating AI into clinical care for the benefit of patients, providers, and healthcare systems, but many challenges remain.

Coordinating the clinical, technical, ethical, and sociotechnical expertise needed to implement impactful AI-based tools is no small feat. However, even when these efforts initially succeed, such tools may face challenges in remaining effective and safe as the performance of the underlying models is disrupted over time by evolving clinical environments.^2^ Patient populations, environmental exposures, clinical care practices, healthcare policies, and patient preferences and care goals can all change over time. Even how we collect patient information shifts, both from a technical perspective and in terms of how we capture information within workflows. This process, referred to as dataset shift or concept drift, influences in predictable and unpredictable ways how well a model trained on previous clinical encounters applies to new patients. As a result, model accuracy deteriorates, reducing utility and potentially leading to safety concerns.^2–5^

Responsible practice necessitates the lifecycle of both analytic and generative AI models extend beyond development, validation, implementation, and impact assessment.^1^^,^^6–9^ If we dedicate the resources to integrate AI into clinical tools and ask both patients and clinicians to trust and rely on these tools, then it is incumbent upon us to ensure they consistently perform as promised. Our work cannot end when we turn a model on, rather that is simply when we enter a new phase of ongoing monitoring and maintenance—a key component of algorithmovigilance.^8^

By default, model maintenance efforts have long relied on complaints from end users. Given the challenge of regaining user trust after perceived model failure and the potential impact on patient care, clinical AI may be more sustainable and successful over time if we can restore struggling models before users are affected. Recent recommendations recognize the role of model governance^3^^,^^9^ and data-driven maintenance methods are expanding.^9–12^ In isolation, however, none of these approaches will be sufficient given the complexity of dataset shift in clinical environments. Some shifts may be intentional and announced, such as software updates or the release of new clinical guidelines. Some, maybe most, will be more nuanced or the unintended consequence of other healthcare and information system priorities. Successfully responding to these varying forces requires algorithmovigilance programs have a suite of tools at their disposal.

In support of healthcare organizations developing model maintenance programs, we propose a 360° continuum of approaches that address model monitoring and maintenance from critically different angles (see Figure 1 and Table 1). We posit that comprehensive algorithmovigilance programs leveraging preventive, preemptive, responsive, and reactive tactics in coordination can sustain clinical AI models, minimize user disruptions, and reliably support patient care.

**Figure 1.**
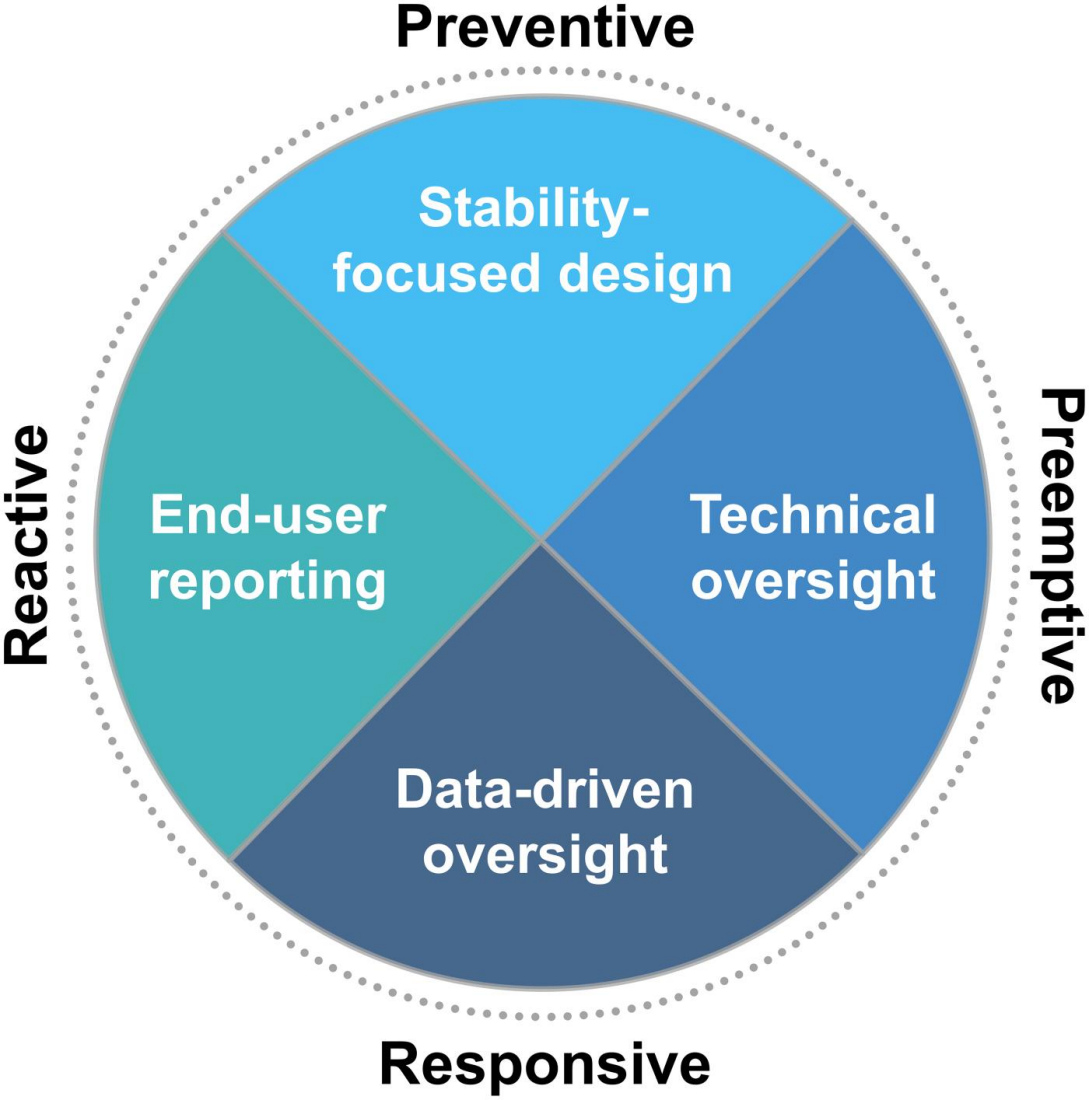
Continuum of algorithmovigilance approaches to ongoing model monitoring and maintenance.

**Table 1.** Overview, advantages, and limitations of perspectives on model monitoring and maintenance.

	Preventive (stability-focused design)	Preemptive (technical oversight)	Responsive (data-driven oversight)	Reactive (end-user reporting)
Approaches	Novel methods aiming to learn stable associations during model training Continuous, dynamic learning algorithms that incorporate dataset shifts as it occurs	Multi-disciplinary team to maintain situational awareness of upcoming technical and clinical changes in order to plan for and address predictable impacts on model performance	Monitor model performance and data inputs; trigger model updating in response to anomalies and trends; recommend and implement updating methods	Users of model predictions notice accuracy issues or diminished utility and report concerns. In response, modeling team investigates and updates or disables models as needed
Benefits	Stability-based models aim to be more transportable, generalizable, and stable in varying environments Continuous models evolve with clinical context and dataset shift May reduce the burden of model management if they successfully maintain performance over longer periods	Can address planned structural changes to inputs (eg, remapping model inputs across coding standards revisions; remapping inputs linked to a deprecated field after system update) Can be responsive to large scale abrupt changes impacting multiple models Enable timely model revision in response to new clinical knowledge or treatment options/guidance Can correct backend technical issues that may manifest as apparent degradation of a model that is still inherently accurate Can proactively tailor models to new clinical sites/populations (eg, local calibration prior to expanded deployment)	Responsive to nuanced and unplanned shifts (eg, gradual changes in outcome prevalence in population) Efficient, timely response to abrupt or gradual shifts (eg, update as needed rather than on a schedule) Focus workload of analytics and knowledge management teams on models most in need of attention (eg, maintain models in background unless performance cannot be restored to clinically acceptable level)	Empowers and engages end users Directly links updates with clinical utility and user needs/expectations May reveal shifts in model utility not directly related to prediction accuracy (eg, workflow changes impacting prediction delivery) Stop-gap measure for abrupt unanticipated changes (eg, missed remapping after system update)
Limitations	Stability-focused methods are still in development and continuous learning represents a paradigm shift in model design These methods will not be able to prevent all performance drift Not all modeling efforts utilize learning algorithms amenable to continuous learning There may be policy, user acceptances, and validation concerns regarding continuous learning models	Requires ongoing review of relevant technical and clinical landscapes by human experts Requires a rigorous system for determining which deployed models may be impacted by each planned change Teams cannot predict all dataset shifts and cannot correct for some shifts until accumulation of sufficient data from the new setting	Statistically significant changes in performance may trigger updating not deemed to be clinically meaningful or necessary Cannot correct for some shifts until accumulation of sufficient data from the new setting May not sufficiently restore performance to acceptable levels which should trigger further review by analytics team or governance body Limited updating approaches possible—Cannot support model revision or extension without user involvement	More burden on busy clinicians to notice and report concerns Possible safety risks before problematic predictions are noticed Need for increased user awareness of the models imbedded in their information systems and understanding of routine maintenance. Loss of user trust (reporter and others) when inaccuracy becomes noticeable, requiring systematic efforts to communicate corrective actions and regain trust

We may be able to *prevent* some model deterioration through careful planning during development. Stability-focused feature selection and learning algorithms minimize model susceptibility to dataset shift.^2^ Such models are expected to be relatively consistent over time and less affected by changing clinical settings. Replacing traditional static models with online, continuous learning models, where appropriate, may also minimize the impact of some dataset shift by actively incorporating new information over time.^12^^,^^13^ However, no models will be robust to all dataset shifts they may encounter and continuous learning models must be scrutinized to ensure errant performance trends do not derail model utility.

We can *preemptively* surveil informatics and clinical landscapes to plan for upcoming technical changes or revisions to clinical guidance. Such technical oversight can allow teams to plan for which—of potentially many—models deployed in their organization may be impacted. These teams could preempt model failures by making backend modifications prior to system updates or initiating necessary revisions to specific models. However, ongoing scrutiny of technical and clinical landscapes is resource intensive, requiring significant expertise and situational awareness. Even well-conducted, complex nuanced dataset shift may not be foreseeable and may defy preemptive measures.

We can be *responsive* to observed deterioration in model accuracy and impact through data-driven surveillance. Running behind the scenes, surveillance systems can actively monitor performance and impact metrics, triggering updating as needed to maintain models in response to unanticipated dataset shift.^10^^,^^11^^,^^14^ While not all updates can be automated and updating may not always restore acceptable performance, responsive data-driven oversight can help sustain multiple models and free up data science teams to concentrate on those models most in need of their intervention.

And of course, we must continue to *react* when end users notice accuracy issues or diminished utility of AI-enabled tools. User feedback may reveal changes unanticipated through technical oversight and not yet detected through data-driven monitoring. User feedback, particularly in coordination with monitoring of process metrics related to model deployments, may also reveal shifts in model utility not directly related to accuracy, such as the need to adjust prediction delivery within clinical workflows. In response, model managers can investigate, update, and even disable models as needed. To sustain user trust and promote stable use of these technologies in healthcare, reactive approaches should be reserved as the mechanism of last resort.

Using this 360° continuum of algorithmovigilance approaches as a conceptual framework may allow healthcare organizations to sustain clinical AI tools more consistently and efficiently, while also limiting the inevitable need for costly, high-resource interventions. Such efforts will require organizational commitment and the establishment of multidisciplinary teams bringing together clinicians, informaticians, data scientists, and health IT professionals.

Preventive and responsive tactics may be led by data scientists that collaborate with clinical champions to tailor model training and updating around clinical requirements. Successful preventive and responsive approaches may minimize periods of instability or inaccuracy; increase maintenance efficiency; aid in prioritizing data science and health IT workloads; and be nearly transparent to end users, helping sustain trust in AI-enabled tools.

Preemptive and reactive tactics may be led by teams of clinicians, informaticians, and health IT professionals that maintain situational awareness of changes both upstream and downstream of model implementations. Consistently scanning the landscape for upcoming changes and investigating end-user concerns may be costly in terms of human resources; however, these approaches are as critical as more automated and less resource-intensive approaches.

Research and policies are needed to develop systems encompassing these tactical perspectives. Practical recommendations for customizing strategies around local resources are also necessary to ensure the benefits of AI-enabled healthcare are available to patients whether they received care at small community hospitals or large academic medical centers. By embracing comprehensive systems for monitoring and maintenance as a priority within our clinical AI deployments and algorithmovigilance programs, we can help ensure the opportunity and value of clinical AI are realized for patients over the long term.

## Data Availability

No new data were generated or analyzed in support of this research.
